# The Treatment of Inflammation, Pain, and Fever Using Medicinal Plants

**DOI:** 10.1155/2012/476985

**Published:** 2012-05-27

**Authors:** Esra Küpeli Akkol, Srijit Das, Satyajit D. Sarker, Lutfun Nahar

**Affiliations:** ^1^Department of Pharmacognosy, Faculty of Pharmacy, Gazi University, 06500 Ankara, Turkey; ^2^Department of Anatomy, Faculty of Medicine, Universiti Kebangsaan Malaysia, Jalan Raja Muda Abd Aziz, 53000 Kuala Lumpur, Malaysia; ^3^Department of Pharmacy, School of Applied Sciences, University of Wolverhampton, Wolverhampton WV1 1LY, UK; ^4^Drug Discovery and Design Research Division, Department of Pharmacy, School of Applied Sciences, University of Wolverhampton, City Campus, MA Building, Wolverhampton WV1 1LY, UK

 As the guest editors of the Journal, Advances in Pharmacological Sciences, we are glad to publish the special issue “The treatment of inflammation, pain, and fever using medicinal plants” receiving enough number of accepted papers. The major drawback of this special issue was* in vivo* and *in vitro* antiinflammatory, antinociceptive, and antipyretic evaluations of the medicinal plants which have ethnobotanical usage. Indeed, natural products have proved to be a rich source of therapeutic agents. Due to the side effects caused mostly by synthetic drugs, interest in natural products is growing rapidly and research into natural products has advanced tremendously in academia and pharmaceutical companies. Therefore, the papers accepted for publication in this special issue provide scientific evidence for the ethnomedicinal features and lead to the development of new drug candidates. For instance, A. Nazrun et al. demonstrated the anti-inflammatory role of vitamin E in prevention of osteoporosis in the research paper entitled “The Anti-inflammatory role of vitamin E in prevention of osteoporosis”. In the study, vitamin E has been shown to inhibit COX-2, the enzyme involved in inflammatory reactions. Of the two types of vitamin E studied, tocotrienol seemed to be better than tocopherol in terms of its ability to suppress bone-resorbing cytokines. In another study by M. Nadia et al., *Labisia pumila *was shown to have phytoestrogenic, anti-inflammatory, and antioxidative properties that make this plant an effective agent against osteoporosis. A mini review by Shilpi et al., was set out to compile and appraise the results on antinociceptive, anti-inflammatory, and antipyretic activity of mangrove plants that grow in the tidal coasts of tropic and subtropic region of the world. This paper finds that antinociceptive, anti-inflammatory, and antipyretic activity appears to be widespread in mangrove plants. According to the research paper by V. Shewale et al. anti-inflammatory activity of *Delonix regia *leaves was studied using carrageenan-induced rat paw edema and cotton pellet granuloma. The ethanol extract of *D. regia *leaves was reported to exhibit significant anti-inflammatory activity. Leaf methanol extract of *C. orbiculata *L. was investigated for antinociceptive and, anti-inflammatory activities using acetic acid writhing, hot-plate tests, and carrageenan-induced edema test by Amabeoku and Kabatende. The data obtained indicated that *C. orbiculata *has antinociceptive and anti-inflammatory activities, justifying the folklore use of the plant species by traditional medicine practitioners in the treatment of painful and inflammatory conditions.

 Taking above mentioned studies into account, Advances in Pharmacology is pleased to publish the special issue “The treatment of inflammation, pain, and fever using medicinal plants”.

*Esra Küpeli Akkol* *Srijit Das* *Satyajit D. Sarker* *Lutfun Nahar*

